# Submicron spatial resolution optical coherence tomography for visualising the 3D structures of cells cultivated in complex culture systems

**DOI:** 10.1038/s41598-021-82178-4

**Published:** 2021-02-10

**Authors:** Chia-Ying Tsai, Cheng-Hung Shih, Hsiao-Sang Chu, Yi-Ting Hsieh, Sheng-Lung Huang, Wei-Li Chen

**Affiliations:** 1 Department of Ophthalmology, National Taiwan University Hospital, Taipei, Taiwan; 2 Graduate Institute of Clinical Medicine, College of Medicine, National Taiwan University, Taipei, Taiwan; 3 Department of Ophthalmology, Fu Jen Catholic University Hospital, Fu Jen Catholic University, New Taipei City, Taiwan; 4 School of Medicine, College of Medicine, Fu Jen Catholic University, New Taipei City, Taiwan; 5 Graduate Institute of Photonics and Optoelectronics, National Taiwan University, Taipei, Taiwan; 6 Department of Ophthalmology, College of Medicine, National Taiwan University, Taipei, Taiwan; 7 Department of Electrical Engineering, National Taiwan University, Taipei, Taiwan; 8 Advanced Ocular Surface and Corneal Nerve Regeneration Center, National Taiwan University Hospital, Taipei, Taiwan

**Keywords:** Corneal diseases, Optoelectronic devices and components

## Abstract

Three-dimensional (3D) configuration of in vitro cultivated cells has been recognised as a valuable tool in developing stem cell and cancer cell therapy. However, currently available imaging approaches for live cells have drawbacks, including unsatisfactory resolution, lack of cross-sectional and 3D images, and poor penetration of multi-layered cell products, especially when cells are cultivated on semitransparent carriers. Herein, we report a prototype of a full-field optical coherence tomography (FF-OCT) system with isotropic submicron spatial resolution in en face and cross-sectional views that provides a label-free, non-invasive platform with high-resolution 3D imaging. We validated the imaging power of this prototype by examining (1) cultivated neuron cells (N2A cell line); (2) multilayered, cultivated limbal epithelial sheets (mCLESs); (3) neuron cells (N2A cell line) and mCLESs cultivated on a semitransparent amniotic membrane (stAM); and (4) directly adherent colonies of neuron-like cells (DACNs) covered by limbal epithelial cell sheets. Our FF-OCT exhibited a penetrance of up to 150 μm in a multilayered cell sheet and displayed the morphological differences of neurons and epithelial cells in complex coculture systems. This FF-OCT is expected to facilitate the visualisation of cultivated cell products in vitro and has a high potential for cell therapy and translational medicine research.

## Introduction

The cell culture model is increasingly valued in medical research, and various complex culture systems and three-dimensional (3D) culture systems are under development^1,2^. Cell culture systems can be used in lieu of experimental animals and provide a large number of consistent and reliable results for biological assays. However, the use of oversimplified in vitro cell assays may limit their clinical value. Recently, the development of a 3D culture system (i.e. tumour spheroid models) in cell therapy research broadened the applicability of cell culture systems in translational medicine and provided novel biological, pathological, and pharmacological information that has dramatically accelerated scientific development^1,2^.

Although the 3D culture system is becoming increasingly popular, a considerable drawback of these complex live cell culture systems is the lack of high-quality monitoring tools. Conventional imaging systems, such as bright-field microscopy and inverted phase-contrast microscopy, are widely used. However, several hindrances that reduce the applicability of these conventional imaging systems should be addressed^3–5^, including inadequate resolution, difficulty in observing cells with multilayer configurations, inability to provide cross-sectional and 3D images, and relatively poor imaging quality obtained through nontransparent matrices or carriers. An optical microscope is limited in resolution and penetration because of its light diffraction characteristics^3^. Far-field fluorescence microscopy techniques, including in vitro confocal and multiphoton microscopy, were developed to improve the spatial resolution and reduce the out-of-focus fluorescence background^4,5^. Other imaging systems used for observing live cells, such as fluorescence microscopy, require additional management of cells that may cause permanent damage to cultivated cells, which is particularly limiting when products are planned for transplantation to patients^6^.

Optical coherence tomography (OCT) is a powerful imaging technique that reveals tissue morphology without additional tissue fixation and labelling. Although researchers have attempted to apply OCT to cultivated cell imaging for label-free usage, the typical axial resolution of conventional OCT, which is usually limited to 5–7 µm, is insufficient to delineate the fine structure within cells^7,8^. Chu et al. used a micro-OCT (µOCT) with lateral and axial resolutions of 2 µm and 1 µm, respectively, to reveal en face and cross-sectional images of dynamic neutrophil transepithelial migration^9^. Huang et al. used OCT to observe 3D tumour spheroid models^7^. Images obtained for tumour spheroids reached approximately 600 µm in height and were expected to have an immediate and long-lasting effect in shortening drug discovery timelines, reducing investment costs and making new medicines available to more patients. Nelson et al. developed optical coherence phase microscopy to assess cell viability and metabolism in 3D tissue culture^10^. Although these techniques are limited, a growing number of papers have demonstrated that this is the right direction for OCT technology for observing live cells.

Our group previously developed a full-field OCT (FF-OCT) system with an isotropic submicron resolution of 0.8–0.9 µm. The resolution improvement in FF-OCT may enable obtaining microstructure images of cultured cells comparable to histopathological findings. We have employed this technique to observe the skin and cornea with satisfactory results^11,12^. Furthermore, we observed blood flow in live animals, which demonstrated the high imaging quality of this system. The en face, cross-sectional, and 3D images obtained using this system demonstrated high imaging resolution. The thin cornea nerves in live animals, which previously could only be seen using in vivo confocal microscopy, were also observed using the en face OCT system^11,13^.

In this study, we used our FF-OCT imaging system to observe various cultivated cells in four complex in vitro cell culture systems. These culture systems are commonly used for corneal research but can be applied to other areas of medical research. First, we demonstrated that cultivated N2A neuron cells and their thin neuron processes could be easily observed using OCT. Second, we observed multilayer cultivated limbal epithelial sheets (mCLESs) prepared for transplantation to treat limbal insufficiency. In this cell culture system, airlifting, a well-documented method for promoting epithelium stratification^1^, was performed following the confluence of the cultivated single layer cell sheet^14–19^. Multilayered cell products prevented the damage of fragile cultivated cells from external stimuli after transplantation to the ocular surface of patients. The cross-sectional and en face imaging quality of our OCT were both satisfactory. Third, we compared the imaging quality of OCT in N2A cells and mCLESs, with and without cultivation on the semitransparent amniotic membrane (stAM). The cell products of mCLESs were widely used in treating total limbal insufficiency, and stAM was widely used as the cell carrier for transplantation^14–19^. The ability of FF-OCT to display the microstructure of cultivated cells through the underneath semitransparent matrix was demonstrated, which solved imaging problems in numerous culture systems in which nontransparent matrix or carriers were needed. Finally, we presented isotropic submicron spatial resolution images of direct adherent colonies of neuron-like cells (DACNs) spread on and covered by limbal epithelial cell sheets in a coculture system. In this culture system, more than two types of cells grew and were mixed and displayed different anatomical relationships in different areas of the same culture dish^2^. The morphology of different DACNs could be clearly identified with our cross-sectional and en face images.

Our OCT could provide real-time images with resolution similar to traditional immunohistochemistry on tissue sections. Therefore, OCT can serve as a promising imaging modality to characterise the morphological features of cells cultivated in complex culture systems. Our results suggest a promising alternative to label-free viability tests for studying live cells under complex culture systems. The results exhibited a high potential for the use of OCT in cell biology, stem cell study, translational medicine, and cell therapy.

## Results

### Imaging quality of neuron cells with delicate neuron processes

**Figure 1:**
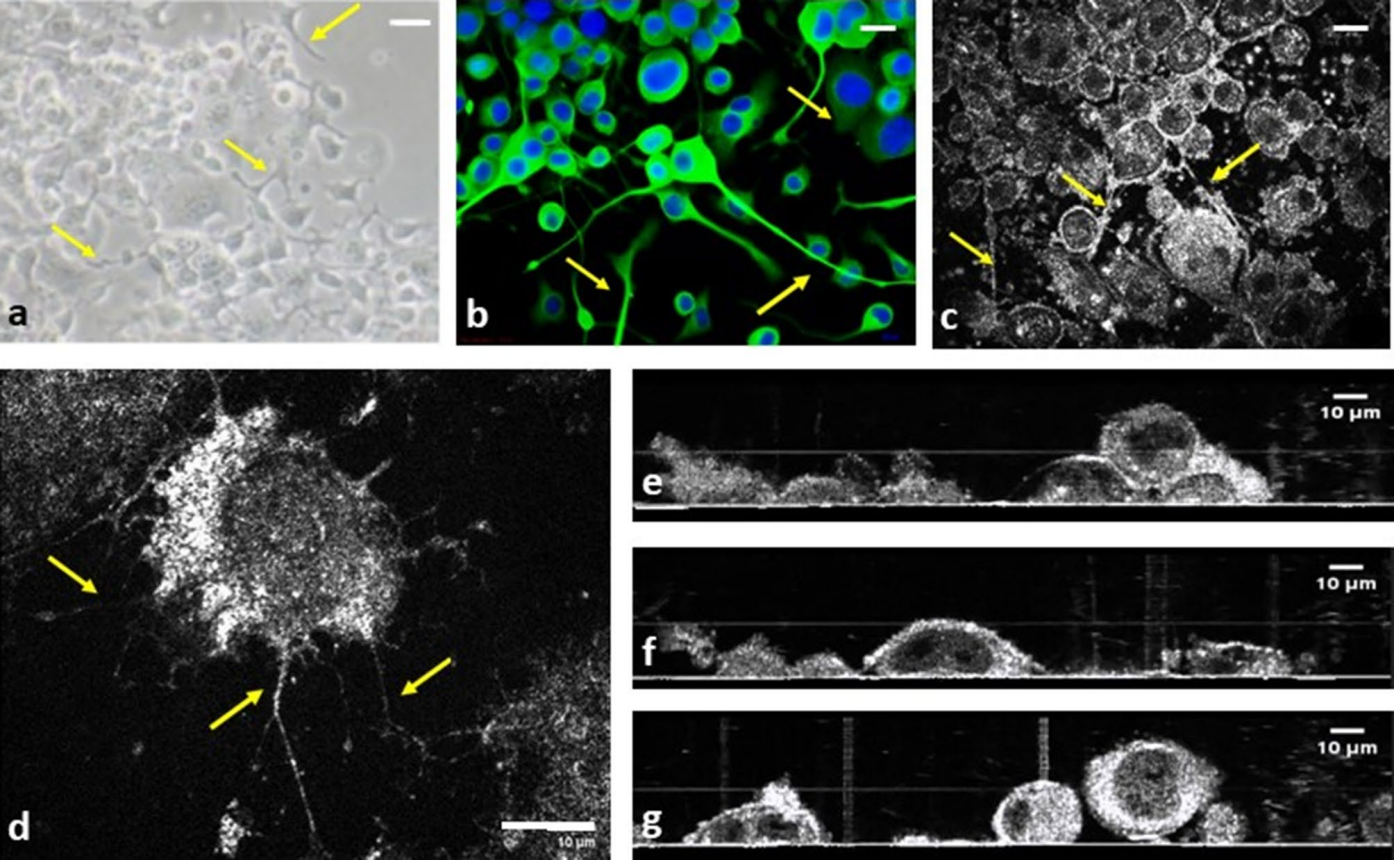
Histocytochemical and FF-OCT images of neuron cells (N2A). (**a**) Phase contrast inverted microscopy revealed the growth patterns of neuron cells. (**b**) Immunocytochemical staining demonstrated a typical growth pattern of neuron cells with long neuron processes. (Green: beta-III tubulin; Blue: Hoechst 33,258 for nucleus). (**c**,**d**) FF-OCT en face images of neuron cells. (**b–d**) Yellow arrows indicated long neuron processes. (**e–g**) Cross-sectional 2D FF-OCT images of neuron cells. The morphology and size of the nucleus and nucleolus in each cell could easily be traced, and more than 1 nucleolus were identified in certain single-cell bodies. Scale bar = 10 µm.

Monolayered cultivated neuron cells (N2A) are displayed in Fig. 1. The phase-contrast image captured using inverted microscopy (Fig. 1a) and immunocytochemistry (ICC) (Fig. 1b), as well as images captured by OCT (Fig. 1c) demonstrated successful cultivation of neuron cells that exhibited typical neuron processes. The long neuron processes were clearly delineated in en face images (Fig. 1c,d). Figure 1e–g demonstrate that the lateral sizes of cell bodies ranged from 10 to 30 μm; the height of the cell body ranged from 9.15 to 28.57 μm, and the nucleus height ranged from 2.80 to 20.00 μm . Furthermore, the morphology and size of the nucleus and nucleoli in each cell could easily be traced with more than one nucleolus identified within certain single-cell bodies (Fig. 1c–g). Additionally, in images captured with inverted microscopy and ICC, the stacked cells lead to difficulty and inaccuracy in counting cell number (Fig. 1a,b). In cross sectional OCT, the stacked cells could be viewed clearly, and makes the cell count more reliable under the assistance of FF-OCT. (Fig. 1e–g).

### Imaging quality of multilayered cultivated cells sheets

**Figure 2:**
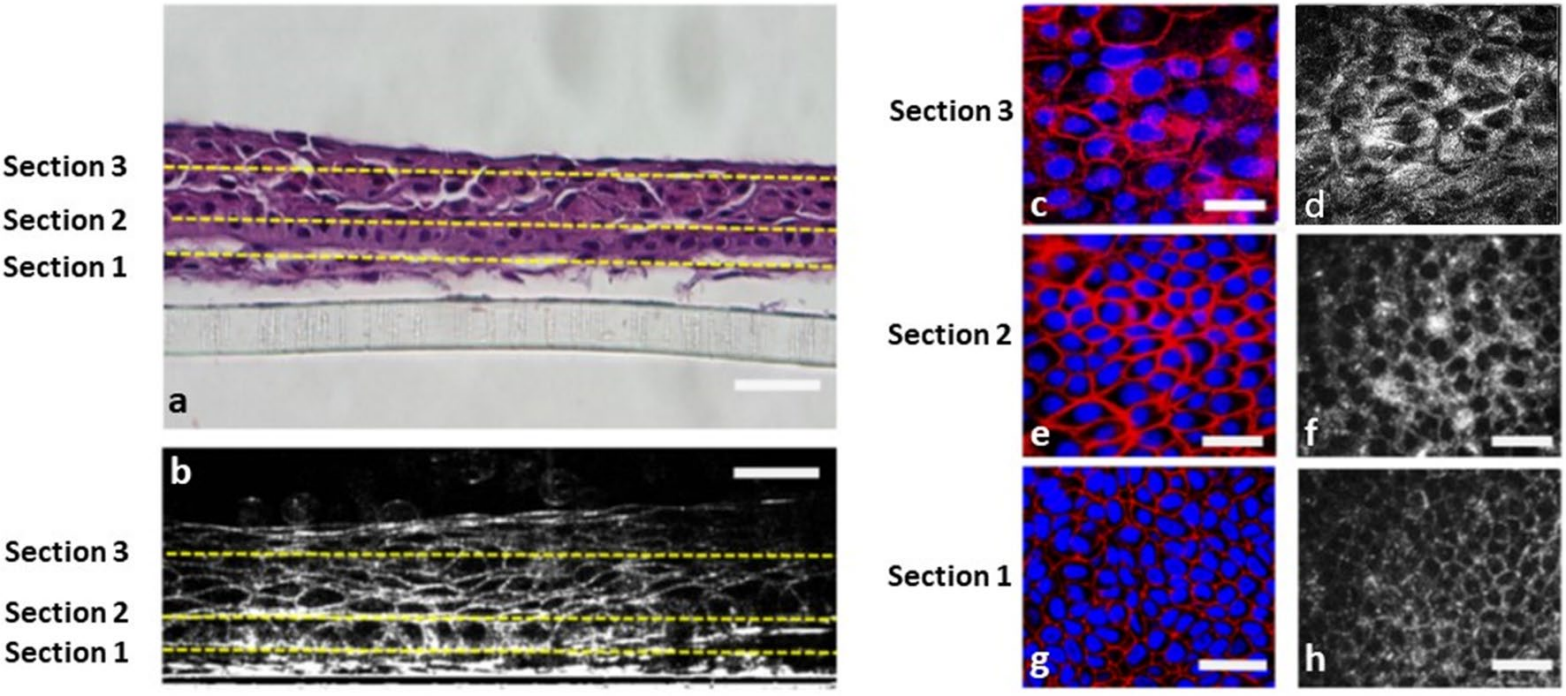
Histological, immunocytochemical, and FF-OCT images of multilayered cultivated cell sheets. (**a**) After the complete culture and formation of mCLESs, haematoxylin and eosin (H&E) staining of the cross-sectional cell product revealed multilayered cell products. (**b**) FF-OCT cross-sectional image of mCLESs. (**a**,**b**) Sections 1, 2, and 3 represent the basal cell layer at 5 µm, wing cell layer at 20 µm, and superficial squamous epithelial layer at 40 µm above the bottom of the culture plate, respectively. (**c**,**e**,**g**) En face images of whole-mount immunocytochemical staining of mCLESs obtained from in vitro confocal microscopy at Section 1 (**g**), Section 2 (**e**), and Section 3 (**c**). Red: Actin. Blue: Hoechst 33,258 for the nucleus. (**d**,**f**,**h**) FF-OCT en face images of mCLESs at Section 1 (**h**), Section 2 (**f**) and Section 3 (**d**). Scale bar = 20 µm. mCLESs = multilayer cultivated limbal epithelial sheets.

Figure 2 represents the cross-sectional and en face images of mCLESs from different layers (section 1 to section 3). As shown in Fig. 2a, the haematoxylin and eosin (H&E) staining of cross-sectional images demonstrated the multilayered structure of the cell product. As depicted in Fig. 2b, the cross-sectional image from our FF-OCT demonstrated the multilayered structure, with clear intercellular borders within each cell. The basal epithelial cells were cuboidal in shape, cells in middle layers were gradually flattened towards outer layers, and superficial cells had a squamous morphology. OCT images revealed the whole thickness and surface evenness of cell products. In certain areas, approximately ten layers of cells could be clearly visualised. Figure 2c–h compares the en face imaging qualities of cells from FF-OCT (Fig. 2d,f,h) and immunocytochemical staining results obtained using in vitro confocal microscopy (Fig. 2c,e,g). Our FF-OCT provided high-quality sequential images captured from different layers that clearly demonstrated the squamous morphology in superficial layers (Fig. 2d), the wing cell morphology in middle cell layers (Fig. 2f), and the cuboidal morphology in basal cell layers (Fig. 2h). We quantified the cell densities in immunocytochemistry (ICC) images and in their corresponding FF-OCT images, and compared the results of these 2 imaging modalities at 3 different layers, namely corneal superficial, wing, and basal layers. The cell densities in ICC images were similar to that in FF-OCT images among all layers, including superficial (ICC: 1160 ± 115.33; OCT: 1150 ± 170.88 cells per millimeter square, *p* = 0.94), wing (ICC: 1987 ± 177.86; OCT: 2080 ± 138.92 cells per millimeter square, *p* = 0.51), and basal layer (ICC: 2960 ± 215.17; OCT: 2973 ± 190.09 cells per millimeter square, *p* = 0.94) (SupplementaryFig. 1a–c). The representative images were shown as Fig. 2c–h. This results support the validity of FF-OCT as an ex vivo tool in measuring cell density.

### Imaging quality of cultivated cells captured from underneath the semitransparent matrix

**Figure 3:**
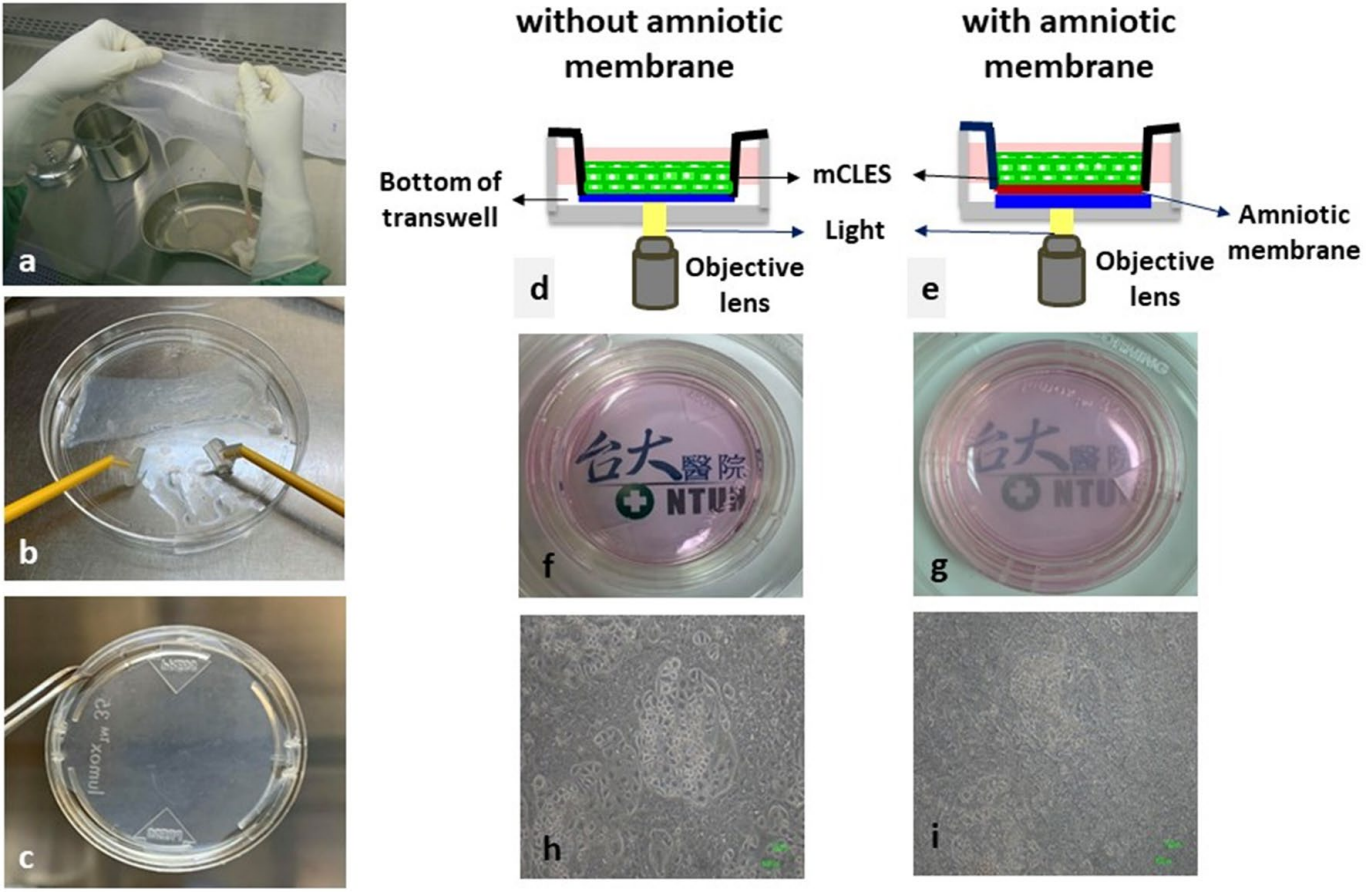
The preparation of the semitransparent amniotic membrane (stAM) as the matrix for cell culture, and the effects of the stAM on inverted microscopy imaging quality impairments. (**a–c**) The harvest of the stAM and the preparation as the matrix for cell culture in a transwell. (**d**,**e**) The spatial relationship of the stAM, bottom of the transwell, cultivated cells, objective lens of the FF-OCT, and light emitted for obtaining images. (**f**,**g**) The stAM was semitransparent and reduced light transmission, and both transwells were added to the culture medium. (**h**,**i**) The stAM reduced the imaging quality of cultivated cells under a phase-contrast inverted light microscope.

**Figure 4:**
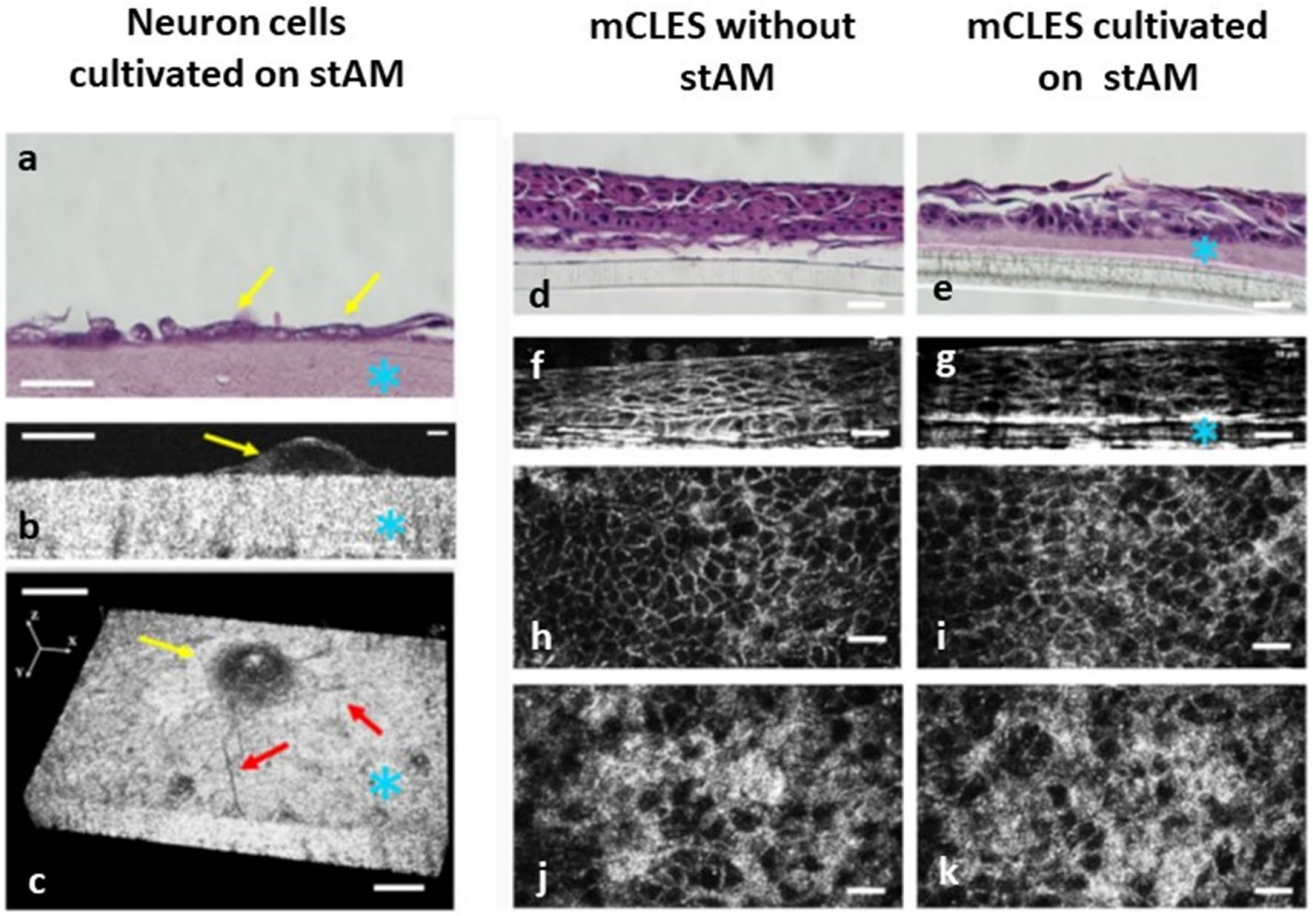
The effects of the stAM on the imaging quality taken by FF-OCT. (**a–c**) Neuron cells (N2A) cultivated on the stAM. (**a**) Hematoxylin and eosin (H&E) staining of cross-sectional neuron cells cultivated on the stAM. Yellow arrow: neuron cells. Blue star: stAM. (**b**) A 2D cross-sectional FF-OCT image of neuron cells cultivated on stAM. Yellow arrow: neuron cells. Blue star: stAM. (**c**) A 3D FF-OCT image of neuron cells cultivated on stAM. Yellow arrow: cell body of the neuron cell. Red arrows: neuron processes. Blue star: stAM. (**d–k**) The effects of the stAM on the FF-OCT cross-sectional and en face imaging quality of cultivated mCLES. (**d**,**e**) H&E staining of the cross-sectional mCLESs with or without stAM as the matrix. Blue star: stAM. (**f**,**g**) A 2D cross-sectional FF-OCT images of mCLESs with or without stAM as the matrix. Blue star: stAM. (**h–k**) Sequential FF-OCT en face images at 5 μm (**h**,**i**) and 30 μm (**j**,**k**) above the bottom of the basal epithelial cells. Scale bar = 20 μm. mCLESs: multilayered cultivated limbal epithelial sheet.

Figure 3 summarises the preparation process of the stAM as the matrix for cell cultures and the effect of the semitransparent matrix on imaging quality. The stAM reduced the transmission ability and imaging quality, both in gross view (Fig. 3f,g) and images of cultivated cells obtained using phase-contrast microscopy (Fig. 3h,i). Figure 4 compares the imaging qualities of N2A cells and mCLESs cultivated with or without the stAM. The light source of FF-OCT was below the culture plate, which enabled clear visualisation of N2A cells and mCLESs through the stAM without interfering with image quality. Figure 4b demonstrates that the thickness of the amniotic membrane was approximately 30 μm, and the height of N2A cells was approximately 15 μm. The 3D FF-OCT image (Fig. 4c) displayed numerous neuron processes emerging from N2A cells cultivated on the amniotic membrane. Supplementary Video 1 and 2 represents en face and transverse video of the neuron process of N2A cells with stAM viewed by our FF-OCT, respectively. These findings demonstrated the ability of this developed OCT to reveal the detailed microstructure of cells through the semitransparent matrix. Figure 4d–k displays the images of mCLESs cultivated with or without the stAM. The cross-sectional OCT images of mCLESs cultured on the stAM exhibited a clear interface between the stAM and cell sheets (Fig. 4g). The en face images of different layers displayed comparable resolutions and qualities between cell products with or without the stAM underneath (Fig. 4h–k).

### The 2D and 3D imaging quality of a complex culture system with different cell types mixed

**Figure 5:**
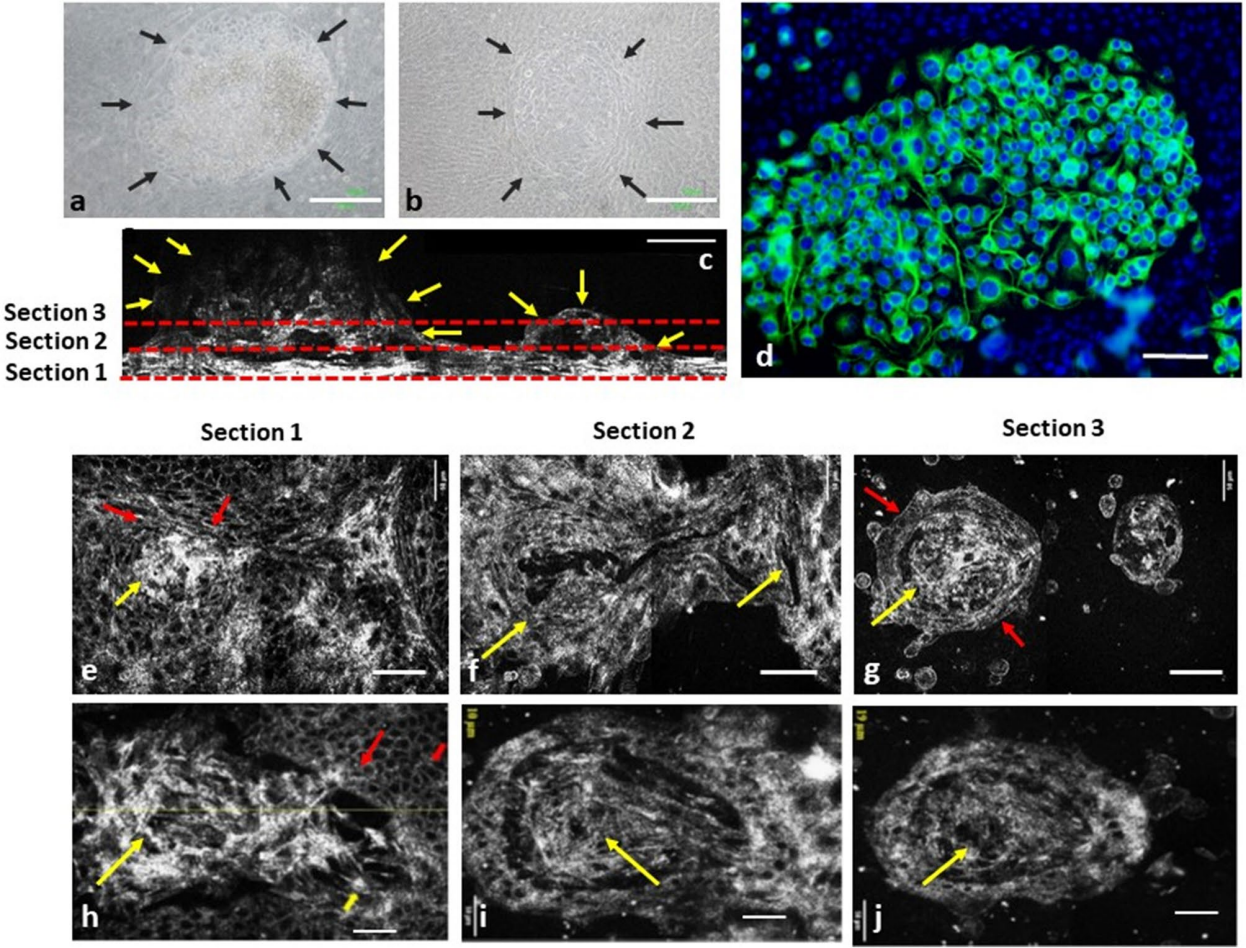
The immunocytochemical, 2D cross-sectional, and en face FF-OCT images of a complex culture system with different types of cells mixed. (**a**,**b**) Phase contrast inverted microscopy demonstrated that two DACNs grew on a sheet of epithelial cells. (**c**) FF-OCT cross-sectional images of the two DACNs. The height of 1 DACN was 150 μm. (**d**) Fluorescein microscopy en face images of the immunocytochemical staining of DACNs (Green: beta-III tubulin; Blue: Hoechst 33258 for nucleus). The staining pattern demonstrated two cell types inside and outside DACNs. (**e–g**) FF-OCT en face image sequence of the culture cells. Section 1 (**e**,**h**), section 2 (**f**,**i**), and section 3 (**g**,**j**) represent the cell at 5 µm, 20 µm, and 50 µm above the bottom of the culture plate. (**e**,**f**,**g**) Sequential images in an area with 2 DACNs illustrated in a single field. Red arrows: epithelial type of cells spread at the bottom of the culture plate, and covered the surface of DACNs. Yellow arrows: cells in the centre of DACNs exhibited different morphologies compared with epithelial cells. (**h**,**I**,**j**) Sequential images from an area with another DACN in a single field. Red arrows: epithelial cells spread at the bottom of the culture plate and covered the surface of DACNs. Yellow arrows: cells in the centre of DACNs exhibited different morphologies to epithelial cells. (**a**,**b**) scale bar = 200 μm, (c-j), scale bar = 50 μm. DACN: directly adherent colonies of neuron-like cells.

**Figure 6:**
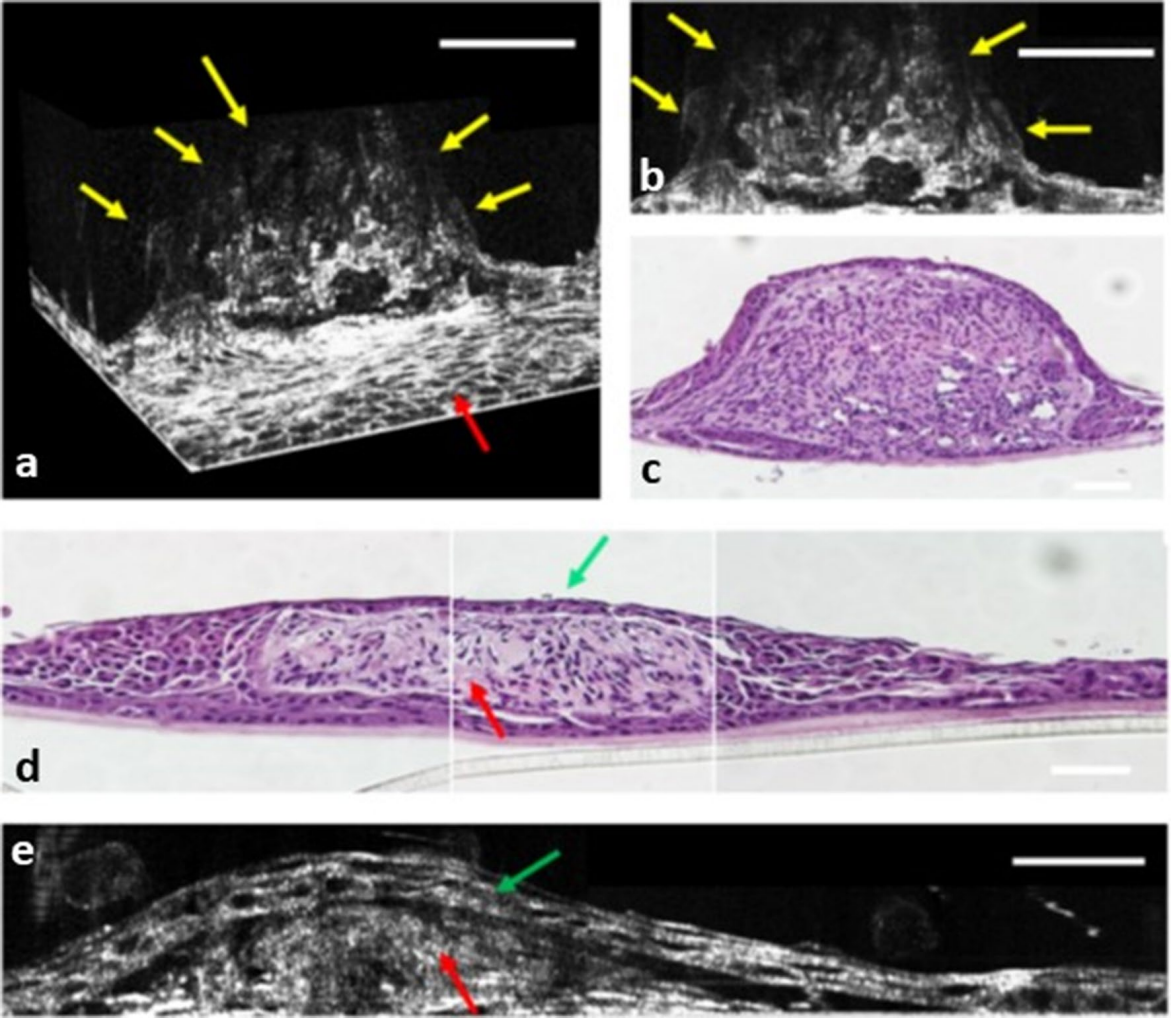
The histological, 3D, and 2D cross-sectional FF-OCT images of multilayered cultivated cell sheets. (**a**) FF-OCT 3D images of DACNs. Yellow arrows indicate the outline surface of DACNs. Red arrows indicate the epithelial type of cells spread at the bottom of DACNs. (**b**) FF-OCT 2D images of DACNs. Yellow arrows indicate the outline surface of DACNs. (**c**,**d**) Hematoxylin and eosin (H&E) staining of cross-sectional DACNs revealed two different cell types, with epithelial cells covering the entire superficial surface of the colonies of neuron-like cells. (**e**) A 2D cross-sectional FF-OCT images of DACNs demonstrated epithelial-like cells covering the surface of neuron-like cells. (**d**,**e**) Green arrows indicated superficial epithelial cells. Red arrows indicated neuron-like cells. (**a**,**b**) Scale bar = 100 μm. (**c**) scale bar = 50 μm. (**d**,**e**) scale bar = 25 μm. DACN: directly adherent colonies of neuron-like cells.

We developed a complex culture system based on a study by Tsai et al.^2^. This study demonstrated that limbal stem cells differentiate into limbal epithelial sheets scatted with DACNs. DACNs are colonies of differentiated neuron-like cells that are covered and scattered on a sheet of limbal epithelial cells. The transverse 2D and en face 2D and 3D images of DACNs are displayed in Figs. 5 and 6 (also see Supplementary Video 3 and 4 online). Figure 5a,b displays two DACNs with different sizes. Figure 5c demonstrates that our FF-OCT can provide clear cross-sectional images of DACNs with a height of up to 150 μm. The sequential en face images of DACNs obtained from different layers along the z (depth) direction at a Z-axis of 5 µm (Fig. 5e,h), 20 µm (Fig. 5f,i), and 50 µm (Fig. 5g,j) from the bottom surface of the culture plate are displayed. The morphology of different cell types and the spatial relationship among different structures were easily identifiable. OCT revealed detailed spatial relationships between different structures that could not be detected through immunocytochemical staining (Fig. 5d). As shown in Fig. 6, the 3D FF-OCT image (Fig. 6a) clearly demonstrated that DACNs grew on a sheet of cells with an epithelial morphology. The 2D and 3D images (Fig. 6a,b) revealed the morphology of DACNs, which was similar to the H&E staining patterns of tissue sections (Fig. 6c). OCT images clearly demonstrated that DACNs consisted of neuron cells covered in a sheet of epithelial cells (Fig. 6d,e). The junction between neurons and epithelial cells was clearly displayed.

## Discussion

In vitro cell culture technology is a valuable research tool that can reduce animal sacrifice, provide reliable and consistent results, and enable the design of various biological conditions. However, the current image detection systems for in vitro cultivated cells have the following disadvantages: (1) Traditional phase-contrast inverted microscopy can only provide en face images, and the resolution is limited because of light diffraction characteristics. Certain subcellular structures are smaller than the wavelength of light, resulting in unsatisfactory resolution at the cellular level^3,20^. Therefore, phase-contrast microscopy cannot be used to image cells stacked into multiple layers or provided 2D cross-sectional or 3D images. An increasing number of cell culture conditions require 3D culture or complex cultured conditions with different cell types mixed, such as the fields of cell regeneration^21,22^, drug discovery^23,24^, and tissue engineering^25,26^; therefore, reliable methods for providing images with high spatial resolution are needed. (2) Traditional histology or immunocytochemistry can provide cross-sectional images. To obtain en face images or 3D images, in vitro confocal microscopy is needed. In all these imaging systems, the cell culturing process has to be discontinued, followed by tissue fixation, sectioning, and staining, which disrupts cell products^6^. Moreover, the real structure and spatial relationships of cells may be destroyed during tissue processing, which is a waste of time and expenses of experiments as well as prevents the long-term observation of the same culture model. (3) Certain cells must be cultured on semitransparent matrices, such as amniotic membranes^15,27,28^. The purpose of these matrices is to provide soluble factors to the extracellular matrix, thus facilitating cell growth. Some semitransparent matrices have been used as carriers of cells cultivated for transplantation^15,29–33^. These live cells, cultured on a semitransparent matrix that reduces certain light transmission, were difficult to visualise during the culturing process. Therefore, ideal imaging systems for monitoring live cultivated cells should have the following characteristics: (1) perform image examination without sacrificing, fixing, sectioning or staining cells; (2) short operation time that facilitates the continued culturing of cells; (3) provide clear and high-resolution images, including cross-sectional, en face, and 3D reconstructed images, that remain clear through a semitransparent matrix; and (4) have a label-free system, so that cells can be repeatedly assessed without labelling-related damages.

In this study, the developed FF-OCT was composed of a crystalline fibre-based light source and a tailored Mirau objective. The former enabled submicron depth resolution with low axial-image-pixel cross talk (AIPCT), because of the broad and near Gaussian spectrum. For in vitro imaging of cell cultures, low AIPCT significantly reduced the ghost image caused by pixel cross talks^34^. The large surface area to volume ratio of the fibre-based light source indicated that heat caused by quantum defects could be effectively removed for high brightness emission, which enables high imaging speed. The specially tailored Mirau objective facilitated the compact interferometer design and eliminated the need for an identical high numerical aperture (NA) objective pair in a conventional Michelson interferometer configuration. Notably, slight structural differences among commercially available high NA objectives, which are caused by manufacturing errors, are manifested at the submicron resolution and result in decreased image quality. Therefore, employing only one objective is advantageous because it fully utilises the light source’s submicron coherence length. The FF-OCT system designed in this study has a unique, fast scanning method for the concurrent display of en face and cross-sectional views with a 3D tomogram. We reported this prototype of the FF-OCT system with isotropic submicron spatial resolution in en face and cross-sectional views and confirmed that it could also provide 3D reconstructed images and a large field of view (FOV) by juxtaposing tomograms^11^. We confirmed the imaging power of this prototype in animals by using in vivo rat and rabbit eyes. We also quantified several anatomical characteristics, such as corneal layer thickness, endothelial cell density, and the intensity profile of different layers. To determine the imaging power of our FF-OCT, we demonstrated that FF-OCT delineated the ridge-like structure of POV, corneal nerve bundles, and conjunctival vessels in rat eyes. The structure of vessel walls and red blood cells in a rabbit model of corneal NV was clearly displayed. The success in animal corneas prompted the application of FF-OCT on cultivated cells. We used the developed FF-OCT to captured the in vitro morphology of various cells, and 2D (en face and cross-sectional) and 3D images were obtained.

**Figure 7:**
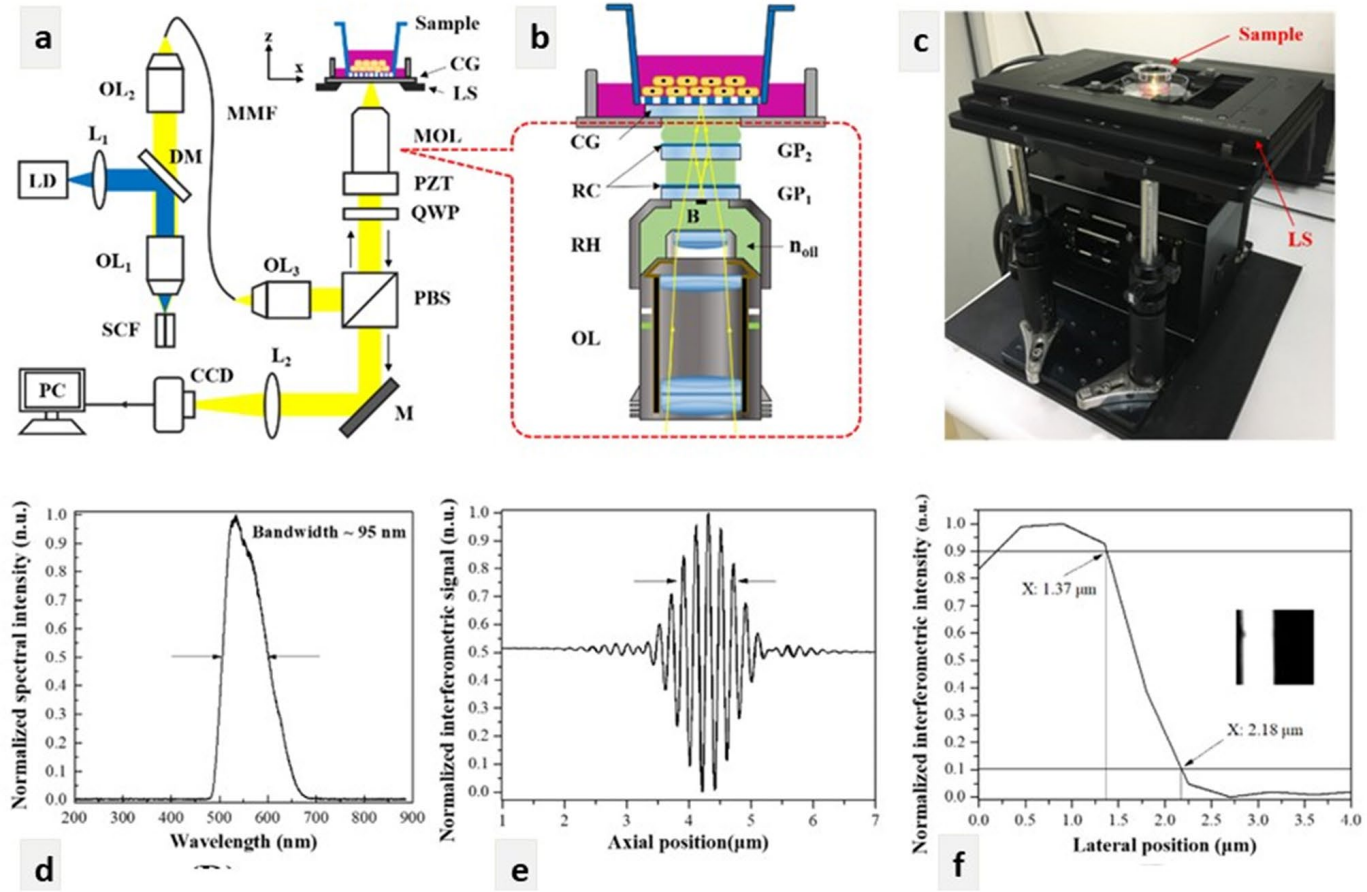
(**a**) Experimental setup of the Mirau-based FF-OCT for cell imaging. LD: 445-nm laser diode; L_1_: 40× aspheric lens; DM: dichroic mirror; OL_1_ and OL_2_: 40× and 20× objective lenses; SCF: single-clad crystal fibre; MMF: multimode fibre (NA: 0.39); OL_3_: 20× objective lens (NA: 0.4); PBS: polarising beam splitter; QWP: quarter-wave plate; PZT: piezo-electric transducer; MOL: home-designed Mirau objective lens; LS: transversal linear stage, CG; cover glass; M: mirror; L_2_: tube lens (focal length: 150 mm); CCD: charge-coupled device. (**b**) Schematic illustration of the MOL with OL: 20× water immersion objective lens; GP_1_ and GP_2_: the first and second glass plates; n_oil_: refractive index of silicon oil (n = 1.406); RC: reflection coating; RH: ring holder; B: 420-µm-diameter black absorber spot. (**c**) Photograph of the Mirau-based FF-OCT. (**d**) The emission spectrum of Ce^3+^:YAG SCF. (**e**) and (**f**) are the axial and lateral resolutions of 0.9 and 0.8 µm, respectively. The inset of (**f**) is the resolution test target imaged by FF-OCT.

We selected several complexes that are widely used in cell culture systems but for which high-resolution images are difficult to obtain using phase-contrast inverted microscopy. FF-OCT provided clear images of neuron cells (Figs. 2 and 5). The thin neuron processes with a width of less than 1 μm, nuclei, and nucleoli (Fig. 2c–g) were clearly imaged, which demonstrated the outstanding resolution of the machine. The cellular system of mCLESs (Fig. 3) mimics most of the multilayered epithelial cells in the body. We used the airlifting technique to stratify cultivated cells into multilayered epithelial cells and then used FF-OCT to obtain 2D cross-sectional and en face images to observe the cell product. We further compared FF-OCT images with cross-sectional H&E staining and the images obtained using immunocytochemistry and in vitro confocal microscopy. We determined that FF-OCT provided images with similar or higher clarity, both in cross-sectional and en face views. A high-quality image series displaying the stratification of mCLESs with different cellular morphologies at different layers provides invaluable information for cell therapy before transplantation. One of the major advantages of FF-OCT is that cell products can be returned to the cell incubator after the examination for further cultivation. Multilayered cell-culture techniques are widely applied in drug development, and mCLESs are widely used for cell therapy in treating patients with limbal insufficiency^14,17,18,32,35–38^; therefore, inspection without harming cells is extremely valuable. We also used cells cultivated with or without stAM to demonstrate that FF-OCT can capture clear images through the semitransparent matrix. Studies regarding the ocular surface frequently employ the stAM, especially when culturing mCLESs^17,18,32,35,37–39^. Our FF-OCT images of cultivated cells were of very high-quality, even with the stAM (Fig. 5). In all cell culture systems, FF-OCT provided a fast, noninvasive, qualitative, and quantitative assessment of cultured cells. The size, layers, shape, growth curve, and intercellular relationships were all clearly demonstrated. To the best of our knowledge, the image resolution obtained from our FF-OCT is higher than other OCT employed to observe cultivated cells^31,39,40^. Finally, we observed DACNs, a special cultivated cell mass developed by our team^2^. The main feature of DACNs is the aggregation of neuron-like cells scattered on and covered by epithelial cell sheets (Figs. 6 and 7). Our FF-OCT provided images in which different types of cells could be clearly distinguished, and the spatial relationship among different types of cells was also clear.

Our FF-OCT system has other optical benefits. First, the light source has a yellow light wavelength, which is safe and can be applied directly to cultivated cells. Second, the handling plate form can be applied to any culturing plate or transwell. However, there are still limitations to our FF-OCT. Our system can provide anatomical information regarding the cell structure but not functional information or biochemical information, which is provided through immunocytochemical staining.

In conclusion, our FF-OCT is a unique and fast scanning method for the concurrent display of en face and cross-sectional views with a 3D display of cultivated cells. This FF-OCT can provide real-time, rapidly obtained, label-free images without harming cultivated cells. This study successfully captured several cell types from complex culture systems. In the future, this imaging modality can be assessed in different cultured cell types.

## Methods

### Mirau-based FF-OCT system and image acquisition

A home-made Mirau-based FF-OCT system with a submicron spatial resolution was employed for biological specimen imaging (i.e. human skin^41^ and cornea^11^). The FF-OCT system was customised to observe cultivated cells (Fig. 7a–c). A double-pass Ce^3+^:YAG single-clad crystal fibre (SCF) with a core diameter of 60 µm and a length of 6 mm was used. The SCF was pumped using a 1-W, 445-nm laser diode to generate the high brightness broadband light source for the FF-OCT system. The 95-nm broadband light output from the SCF with a centre wavelength of 560 nm (Fig. 7d) was then delivered to the FF-OCT system by using a multimode fibre (400-µm core, NA: 0.39, Thorlabs, Newton, NJ). Thus, the FF-OCT system had an axial resolution of 0.9 µm in vitro (Fig. 7e). As illustrated in Fig. 7b, a 20× water-immersion objective (UMPlanFLN 20× W, NA: 0.5, Olympus, Tokyo, Japan) was adopted in the custom-designed Mirau objective lens to achieve near-isotropic spatial resolution. A high lateral system resolution of 0.8 µm was verified using the interferometric intensity of the test target (Fig. 7f). The Mirau-based OCT configuration had an inherently chromatic dispersion compensation from the specimen and was prone to reduce noise caused by environmental vibration.

The images of FF-OCT are created by acquiring multiple en face images by using a 400-µm-long piezo-electric transducer (PZT) (PI, #P-725.40L) scanning along the z (depth) direction. The FOV of each tomogram was 292 × 220 µm (x × y), and the pixel size was 0.45 × 0.45 µm. The PZT scanning speed was ≤ 13 µm/s, and the OCT en face frame rate of 65 frames/s was in accordance with CCD’s (#ICL-B0620, 648 × 488 pixels, Imperx, Boca Raton, FL) frame rate of 260 frames/s. The interference fringe was demodulated using the 4-point method with 2–6/point of signal depending on the strength of the backscattered light from the sample. The tomograms obtained from the OCT system were stored as en face stack frames with 0.2-µm separation.

The cultivated cells were imaged from the interface between a specially tailored culture dish and the bottom surface of cells or from underneath the amniotic membrane. The culture dish bottom plate was composed of fused silica so that the optical dispersion of the FF-OCT sample arm matched the optical dispersion of the reference arm. The transversal linear stage (MLS203-1, Thorlabs) was equipped to assess a large area by stitching adjacent tomograms for a deeper understanding of cultivated cells. The maximum available FOV of the system was 1 × 1 mm (x × y). Image processing was applied to each original OCT stack image by using the mean 3D plugin in ImageJ. The mean 3D processing was performed by averaging pixels within a set radius of the spherical volume. Furthermore, the ImageJ plugin 3D viewer was applied to reconstruct volumetric images.

### Animals

New Zealand albino rabbits (3.0–3.5 kg and 6 months old) were used in this study. Treatment protocols for all animals followed the regulations of the Association for Research in Vision and Ophthalmology Statement for the Use of Animals in Ophthalmic and Vision Research (IACUC approval No.: 20160089). Furthermore, all experimental procedures were approved by the Committee for Animal Research of National Taiwan University Hospital. The rabbits were euthanised with injected overdosed potassium chloride (5 mL/kg) after intravenous injection of 240 mg/kg thiamylal sodium (Shinlin Singseng Pharmaceutical, Taoyuan, Taiwan) through the marginal ear vein before tissue samples for cell cultures were collected.

### stAM harvesting and preparation

The stAM was peeled from the human donated placental tissue after elective cesarean delivery and processed under sterile conditions by the tissue bank of National Taiwan University Hospital. The stAM was carefully attached and fixed to the upper well of the transwell (Corning, Inc., Corning, NY) and was air-dried for 8 h before adding the culture medium. The stAM was used as the carrier for the cultivation of neuroblastoma cell lines (N2A, Neuro2A, ATCC) or mCLES form of cell therapy.

### Culture of neuron cells

To evaluate the ability of OCT to image neuron cells with delicate neuron processed and the effect of stAM on the resolution ability of OCT, the culture technique of the N2A cell line was modified^2^. The neuroblastoma cell line (N2A) was cultivated using N2A medium (growth medium of ATCC-formulated Eagle’s Minimum Essential Medium [EMEM] and 10% FBS) with or without fixing the stAM on the upper well of transwells as matrix and keeping in room air for dryness for 8 h. The medium was changed every 2 days.

### Cell-culture model of mCLESs

To evaluate the ability of OCT to study multilayered cell products and the possible effect of stAM on the resolution ability of OCT, the culture technique of mCLESs was modified from our previous study^1^. In brief, limbal stem cells were extracted from the limbal area of adult New Zealand rabbits after euthanasia. After enucleation of the eyeballs, the limbal tissue, preserved at 1 mm within and beyond the limbal area, was cut into 12 equal pieces after a gentle wash with phosphate-buffered saline (PBS). The samples were treated with 1 mg/mL collagenase A (Roche, Indianapolis, IN) supplemented with hormonal epithelial medium at 37 °C for 18 h. The stromal components were fully digested, and the remaining limbal epithelial sheet was transferred with a pipette to the upper part of transwells, with or without stAM coating. The culture medium was composed of supplemented hormonal epithelial medium comprising Dulbecco modified Eagle medium/Ham F-12 nutrient mixture (1:1 ratio; Thermo Fisher Scientific Cell Culture, Portland, OR), which contains 5 mg/mL insulin (Thermo Fisher Scientific, Waltham, MA), 1 nM cholera toxin (Sigma-Aldrich Co., St. Louis, MO, USA), 10 ng/mL epidermal growth factor (Thermo Fisher Scientific), 0.5% dimethyl sulfoxide (Sigma-Aldrich), and 0.5 mg/mL hydrocortisone (Sigma-Aldrich), supplemented with 5% foetal bovine serum (Thermo Fisher Scientific). The medium was placed for 24 h at 37 °C under 5.0% CO_2_ to facilitate cluster attachment. The medium was then changed every 2 days after the attachment of cell clusters and then spread of the cell into a confluent sheet. After cells became confluent, airlifting was performed for 24 h to trigger cellular stratification^1^, thereby forming mCLESs.

### A complex culture system for studying different cell types mixed: The growth of directly adherent colonies of neuron-like cells scattered on and covered by limbal epithelial cells sheets

A system to observe the multipotency of limbal stem cells was developed. Limbal stem cells have been demonstrated to differentiate into DACNs scattered on and covered by limbal epithelial cell sheets in a coculture system^2^. In the lower well of this coculture system, N2A cells coated with 10 μg/mL fibronectin (Sigma-Aldrich) were cultivated using transwell (Corning). In the upper well of this coculture system, limbal stem cells were extracted from the limbal area. Retinoid acid (Sigma-Aldrich) (20 μM) was added to the N2A medium (growth medium of ATCC-formulated EMEM and 10% FBS to trigger neuronal differentiation). Our indirect coculture system was designed to cultivate limbal tissue and N2A cell lines on different sides of a transwell. Our previous result indicated that DACN appeared and grew consistently within 3 days, and it continuously grew, scattered on the sheet layer of LES. Cell growth patterns were observed using an inverted microscope equipped with a camera. The observation of DACNs on a sheet layer of LES was performed using FF-OCT after removing upper wells from transwells.

### Immunocytochemical staining examined using fluorescence microscopy

In the first step of immucocytochemial staining, we fixed the neuron cells with 4% paraformaldehyde after washing them with PBS, and then blocked the cells with 1% bovine serum albumin. In following steps, N2A cells and DACNs were incubated with primary antibody (1:100 anti-beta III tubulin, Abcam, Cambridge, UK) overnight at 4 °C, and then with FITC-conjugated secondary antibodies (Life Technologies, Carlsbad, CA) (1:100). We counterstained the nuclei with Hoechst 33258 (Invitrogen.

Life Technologies, Carlsbad, CA), and then mounted the cells. Eclipse E800 microscope with a VFM epi-fluorescence attachment (Nikon, Melville, NY) was used for observing the stained cells, and the pictures were taken by the SPOT digital camera with SPOT version 1.1 CE software (Diagnostic Instruments, Sterling Heights, MI) attached to the microscope. All experiments were repeated for three times.

### Whole-mount immunocytochemical staining imaged by in vitro confocal microscopy

The mCLESs for histological examination were embedded in an optimal cutting temperature compound and cut into frozen sections of 8-μm thickness. The sections were then stained using H&E. For the whole-mount immunocytochemical staining with in vitro confocal microscopy, mCLESs were fixed with acetone and subsequently permeabilised and blocked using 0.1% Triton X-100 and 2% goat serum. The samples were incubated with anti-actin (Zymed, San Francisco, CA). The cell sheets were then rinsed and incubated with propidium iodide-conjugated secondary antibodies. The nuclei were counterstained using Hoechst 33258 (Invitrogen Life Technologies). Negative controls were obtained by omitting the primary antibody. Cell sheets were then mounted and examined using in vitro confocal microscopy. Z-stack images were captured in 1-μm sections from the apical cell layer to the basal cell layer. The images captured were analysed using Zen software. A Leica TCS SP2 confocal microscope was used for imaging.

### Quantification of cell density in the cultivated multi-layered cell sheet

Cell densities in corneal superficial, wing, and basal layers were calculated by averaging the measured cell densities of three different areas in each layer in the confocal microscopic images. The corresponding FF-OCT images focusing on the same area from each layer of the cultivated cell sheet were also collected and measured for the cell densities. Images were analyzed using ImageJ software (National Institute of Health; Bethesda, MD, USA) to delineate individual cells and facilitate cell counting.

### Statistical analysis

Descriptive statistics for variables are reported as mean ± SD and analyzed using SPSS software, version 19.0 (SPSS Inc., Chicago, IL). Differences between the means of cell density from different imaging modality were analyzed using the Mann–Whitney *U* test. A *p* value less than 0.05 was considered statistically significant.

## Supplementary Information

Supplementary Information 1.

Supplementary Video 1.

Supplementary Video 2.

Supplementary Video 3.

Supplementary Video 4.

Supplementary Information 2.
