# Supplementary material for: Submicron spatial resolution optical coherence tomography for visualising the 3D structures of cells cultivated in complex culture systems
Source: Sci Rep. 2021 Feb 10;11:3492. doi: 10.1038/s41598-021-82178-4 (PMC7875968; doi:10.1038/s41598-021-82178-4)
Supplement: Supplementary file 1 — Supplementary Information 1. [file 41598_2021_82178_MOESM1_ESM.docx]

**Supplementary Information**

Title:

Submicron Spatial Resolution Optical Coherence Tomography for Visualising the 3D Structures of Cells Cultivated in Complex Culture Systems

Author list:

Chia-Ying Tsai, MD^1,2,3,4^, Cheng-Hung Shih, MS^5^, Hsiao-Sang Chu^1,2,6^, Yi-Ting Hsieh^1^, Sheng-Lung Huang^5,7*^, Wei-Li Chen, MD, PhD ^1,6,8*^,

1. Department of Ophthalmology, National Taiwan University Hospital, Taipei, Taiwan
2. Graduate Institute of Clinical Medicine, College of Medicine, National Taiwan University, Taipei, Taiwan
3. Department of Ophthalmology, Fu Jen Catholic University Hospital, Fu Jen Catholic University, New Taipei City, Taiwan
4. School of Medicine, College of Medicine, Fu Jen Catholic University, New Taipei City, Taiwan
5. Graduate Institute of Photonics and Optoelectronics, National Taiwan University, Taipei, Taiwan
6. Department of Ophthalmology, College of Medicine, National Taiwan University, Taipei, Taiwan
7. Department of Electrical Engineering, National Taiwan University, Taipei, Taiwan.
8. Advanced Ocular Surface and Corneal Nerve Regeneration Center, National Taiwan University Hospital, Taipei, Taiwan.

*Co-corresponding author

Commercial relationships: None

Address correspondence: W.-L.C. (email: email: chenweili@ntu.edu.tw), S.-L.H. (email: shuang@ntu.edu.tw)

**Supplementary Video 1**: En face video of the neuron process of neuron-2A (N2A) cells with semitransparent amniotic membrane (stAM) viewed by our full-field optical coherence tomography (FF-OCT)

**Supplementary Video 2**: Transverse video of the neuron process of neuron-2A (N2A) cells with semitransparent amniotic membrane (stAM) viewed by our full-field optical coherence tomography (FF-OCT)

**Supplementary Video 3**: The en face video of directly adherent colonies of neuron-like cells (DACNs)

**Supplementary Video 4**: The transverse video of directly adherent colonies of neuron-like cells (DACNs)

**Supplementary Figure 1:** The comparison of cell densities in immunocytochemistry (ICC) images and in the corresponding FF-OCT images from superficial, wing, and basal layer of multilayered cultivated cell sheets.
