# Supplementary figures and images for: Submicron spatial resolution optical coherence tomography for visualising the 3D structures of cells cultivated in complex culture systems
Source: Sci Rep. 2021 Feb 10;11:3492. doi: 10.1038/s41598-021-82178-4 (PMC7875968; doi:10.1038/s41598-021-82178-4)

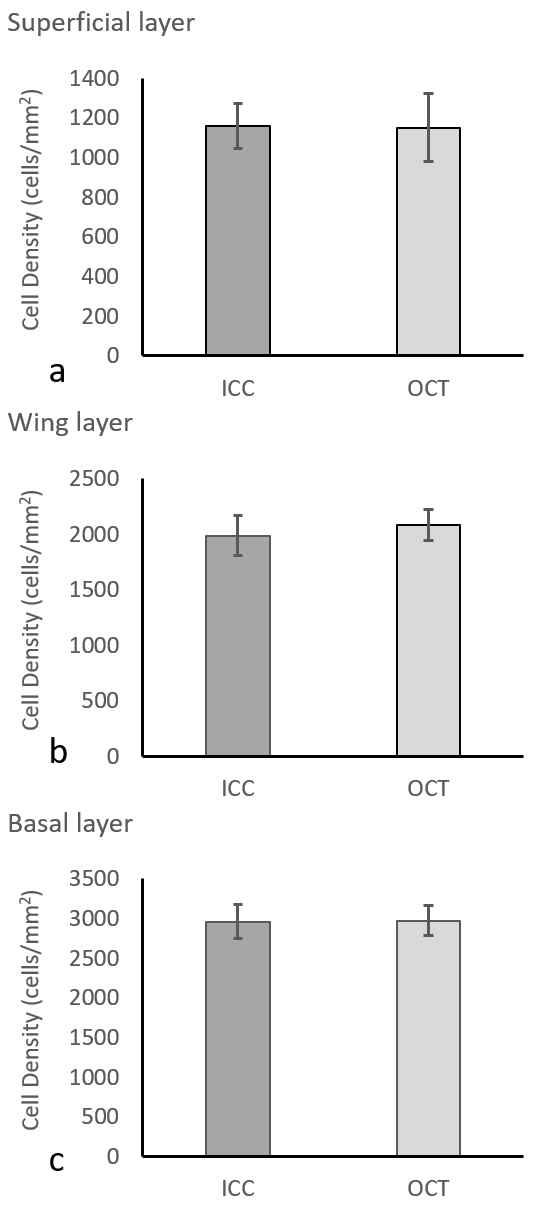

Supplement: Supplementary file 6 — Supplementary Information 2. [file 41598_2021_82178_MOESM6_ESM.tif]
